# Characteristics of *Salmonella enterica* Serovar 4,[5],12:i:- as a Monophasic Variant of Serovar Typhimurium

**DOI:** 10.1371/journal.pone.0104380

**Published:** 2014-08-05

**Authors:** Noriko Ido, Ken-ichi Lee, Kaori Iwabuchi, Hidemasa Izumiya, Ikuo Uchida, Masahiro Kusumoto, Taketoshi Iwata, Makoto Ohnishi, Masato Akiba

**Affiliations:** 1 Iwate Prefecture Central Livestock Hygiene Service Center, Iwate, Japan; 2 Bacterial and Parasitic Disease Research Division, National Institute of Animal Health, National Agriculture and Food Research Organization, Ibaraki, Japan; 3 Research Institute for Environmental Sciences and Public Health of Iwate Prefecture, Iwate, Japan; 4 Department of Bacteriology, National Institute of Infectious Diseases, Tokyo, Japan; 5 Hokkaido Research Station, National Institute of Animal Health, National Agriculture and Food Research Organization, Hokkaido, Japan; 6 Graduate School of Life and Environmental Sciences, Osaka Prefecture University, Osaka, Japan; University of Saskatchewan, Canada

## Abstract

*Salmonella enterica* subspecies *enterica* serovar 4,[5],12:i:- (*S*. 4,[5]12:i:-) is believed to be a monophasic variant of *S*. *enterica* serovar Typhimurium (*S*. Typhimurium). This study was conducted to corroborate this hypothesis and to identify the molecular and phenotypic characteristics of the *S*. 4,[5]12:i:- isolates in Japan. A total of 51 *S*. 4,[5]12:i:- isolates derived from humans, cattle, swine, chickens, birds, meat (pork), and river water in 15 prefectures in Japan between 2000 and 2010 were analyzed. All the *S*. 4,[5],12:i:- isolates were identified as *S*. Typhimurium by two different polymerase chain reactions (PCR) for identification of *S*. Typhimurium. Of the 51 *S*. 4,[5],12:i:- isolates, 39 (76.5%) harbored a 94-kb virulence plasmid, which is known to be specific for *S*. Typhimurium. These data suggest that the *S*. 4,[5],12:i:- isolates are monophasic variants of *S*. Typhimurium. The flagellar phase variation is induced by three adjacent genes (*fljA*, *fljB*, and *hin*) in the chromosome. The results of PCR mapping of this region and comparative genomic hybridization analysis suggested that the deletion of the *fljAB* operon and its flanking region was the major genetic basis of the monophasic phenotype of *S*. 4,[5],12:i:-. The *fljAB* operon and *hin* gene were detectable in eight of the *S*. 4,[5],12:i:- isolates with common amino acid substitutions of A46T in FljA and R140L in Hin. The introduction of these mutations into *S*. Typhimurium isolates led to the loss of selectability of isolates expressing the phase 2 H antigen. These data suggested that a point mutation was the genetic basis, at least in part, of the *S*. 4,[5],12:i:- isolates. The results of phenotypic analysis suggested that the *S*. 4,[5],12:i:- isolates in Japan consist of multiple distinct clones. This is the first detailed characterization of the *S*. 4,[5],12:i:- isolates derived from various sources across Japan.

## Introduction

Nontyphoidal salmonellae are one of the most common cause of bacterial gastroenteritis in humans as well as salmonellosis in domestic and wild animals worldwide [1]. Serotyping is widely used as an epidemiological typing method to subdivide *Salmonella* species [2]. Each serovar is identified by the combination of lipopolysaccharide moieties on the cell surface (O antigens) and one or two different flagellar proteins (H antigens). Many serovars have the ability to express two different flagellin proteins, although individual cells can express one of the two flagellins [3]. According to the White-Kaufmann-Le Minor scheme, more than 2500 serovars to date have been recognized in the genus *Salmonella* [4].

The incidence of human salmonellosis caused by *Salmonella enterica* subspecies *enterica* serovar 4,[5],12:i:- (*S*. 4,[5],12:i:-) has been increasing in Europe, North and South America, and Asia since the mid-1990s [5]–[9]. This serovar is currently among the 10 most common serovars responsible for human infections in a variety of countries, including the second and sixth most prevalent serovar in Germany [10] and the United States [11], respectively. *S*. 4,[5],12:i:- was also reported as the fourth most common serovar in slaughtered pigs in the EU [10]. In Japan, the rate of distribution of this serovar was more than 2% (ninth most prevalent) in 2009 for the first time, and then remained relatively high to date [12]. Larger outbreaks caused by this serovar have been reported in the United States and Luxemburg [13], [14].

*S*. 4,[5],12:i:- does not appear in the White-Kaufmann-Le Minor scheme [4] and appears to be a monophasic variant of other biphasic serovars, which have lost phase 2 flagellin or the necessary switching mechanism of phase variation. Seven serovars of *S*. *enterica* subsp. *enterica* with same O and phase 1 H antigens are possible ancestors of this serovar, including Typhimurium (*S*. Typhimurium), Lagos, Agama, Farsta, Tsevie, Gloucester, and Tumodi [4]. Among these, *S*. Typhimurium is commonly isolated from humans, animals, and the environment, whereas the others are rarely isolated.

*S*. Typhimurium is believed to be an ancestor of *S*. 4,[5],12:i:- based on the following evidence. *S*. Typhimurium-specific sequences have been detected in *S*. 4,[5],12:i:- [15]. Some of the *S*. 4,[5],12:i:- isolates showed the same lysogenic patterns as those of *S*. Typhimurium by phage typing and displayed pulsed-field gel electrophoresis patterns identical or similar to those of *S*. Typhimurium isolates [15]–[18]. Different deletions and mutations can be responsible for the lack of phase 2 flagellin expression among the *S*. 4,[5],12:i:- isolates. Specifically, some isolates from Spain lack 16 genes, including the *fljAB* operon and flanking genes, which encode phase 2 flagellin expression-related proteins [19]. Some of the *S*. 4,[5],12:i:- isolates from the United States appeared to have smaller deletions or point mutations in the *fljAB* operon and/or flanking genes not identified by deoxyribonucleic acid (DNA) probes [18]. To date, no specific point mutations affecting the phase 2 flagellin expression have been identified.

In the present study, we characterized the *S*. 4,[5],12:i:- isolates derived from various sources in Japan for the following purpose: (i) to corroborate the hypothesis that the *S*. 4,[5],12:i:- isolates are monophasic variants of *S*. Typhimurium, (ii) to elucidate the genetic basis of the monophasic phenotype of the *S*. 4,[5],12:i:- isolates, (iii) to identify the molecular and phenotypic characteristics of the *S*. 4,[5],12:i:- isolates in Japan because only limited information is currently available.

## Materials and Methods

### Bacterial isolation, identification, and typing

The *S*. 4,[5],12:i:- isolates used in this study are listed in Table 1. A total of 51 isolates were derived from humans, cattle, swine, chickens, birds, meat (pork), and river water from 15 prefectures in Japan between 2000 and 2010. The isolates from humans and cattle were obtained from fecal samples of patients or affected animals with different sporadic infections. The swine isolates were obtained from fecal samples of healthy or affected animals. The isolates from chickens and crows were obtained from fecal samples or organs of healthy birds. The isolates from a penguin and a parrot were obtained from organs of diseased birds. The isolates from pork and river water were obtained from a previous monitoring study. *S*. Typhimurium strain LT2 [20] was used as a positive control for polymerase chain reaction (PCR) analysis or as a reference for comparative genomic hybridization analysis, and strains L-3900 and L-3287 were used to introduce point mutations identified among the *S*. 4,[5],12:i:- isolates by gene replacement. In Japan, L-3900 was isolated from cattle in 2010, whereas L-3287 was isolated from chicken in 2001. Both these strains were susceptible to kanamycin. The isolation of *S. enterica* was performed by the staff of the local Institute of Public Health or local Animal Hygiene Service Centers for diagnostic or monitoring purposes. Patient information was anonymized and de-identified prior to analysis. The approval from the Institutional Animal Care and Use Committee is not required in case of isolation for diagnostic purpose. Isolates H1–5 and C1–10 were the same as H1–5 and C1–10, respectively, as described in a previous report [7]. *Salmonella* spp. were identified based on colony morphology on selective media and biochemical properties, as previously described [21]. Serovar identification was performed by microtiter and slide agglutination methods according to the latest version of the White-Kaufmann-Le Minor scheme [4] using antiserum (Denka Seiken Co., Ltd., Tokyo, Japan). Phage typing was performed using *S*. Typhimurium typing phages according to the methods and schemes previously described by Anderson et al. [22]. All isolates were maintained at −80°C in Luria–Bertani (LB) broth (Becton, Dickenson and Company, Sparks, MD, USA) containing 25% (v/v) glycerol.

**Table 1:** *Salmonella enterica* serovar 4,[5],12:i:- isolates used in this study.

			PCR results^a^											
Isolates	Source	Year	m-PCR	IS*200*	up-*fljA*	*fljA-fljB*	*fljB-hin*	*hin*-down	*fin*	*spvB*	94 kb plasmid^b^	Phage type^c^	LDC^d^	Resistance profile^e^
H1∼4	Human	2006	+	+	-	-	-	+	-	+	+	193	+	-
H5	Human	2007	+	+	-	-	-	-	-	-	-	193	+	ASSu
H6	Human	2008	+	+	-	-	-	+	-	+	+	RDNC-a	+	-
H7	Human	2003	+	+	-	-	-	+	-	+	+	193	+	-
H8	Human	2007	+	+	+	+	+	+	-	+	+	26	+	-
H9∼11	Human	2007	+	+	-	-	-	+	-	+	+	RDNC-a	+	-
H12	Human	2004	+	+	-	-	-	+	-	-	-	RDNC-c	+	-
H13	Human	2007	+	+	-	-	-	-	-	-	-	193	+	SSuT
H14	Human	2002	+	+	-	-	-	-	-	+	+	UT	+	ASuT
C1	Cattle	2003	+	+	-	-	-	+	-	+	+	RDNC-a	-	-
C2	Cattle	2005	+	+	-	-	-	+	-	+	+	RDNC-a	-	-
C3∼4	Cattle	2007	+	+	-	-	-	+	-	+	+	RDNC-a	-	-
C5∼8	Cattle	2008	+	+	-	-	-	+	-	+	+	RDNC-a	-	-
C9∼10	Cattle	2008	+	+	-	-	-	-	-	+	+	RDNC-a	+	A
C11	Cattle	2004	+	+	-	-	-	+	-	+	+	RDNC-a	+	-
C12	Cattle	2005	+	+	-	-	-	+	-	+	+	120	+	-
C13	Cattle	2005	+	+	+	+	+	+	-	+	+	RDNC-b	+	-
C14	Cattle	2008	+	+	-	-	-	-	-	-	-	UT	+	ASSuT
C15	Cattle	2007	+	+	-	-	-	+	-	+	+	RDNC	+	-
C16	Cattle	2010	+	+	-	-	-	+	-	+	+	RDNC-a	+	-
C17	Cattle	2010	+	+	-	-	-	+	-	+	+	RDNC-b	+	A
S1	Swine	2008	+	+	-	-	-	-	-	-	-	UT	+	ASSuT
S2	Swine	2009	+	+	-	-	-	-	-	-	-	UT	+	ASSu
S3	Swine	2002	+	+	-	-	-	-	-	-	-	RDNC-d	+	Ssu
S4	Swine	2003	+	+	-	-	-	-	-	-	-	RDNC-d	+	SSuT
S5	Swine	2008	+	+	-	-	-	-	-	-	-	193	+	SSuT
S6	Swine	2009	+	+	-	-	-	-	+	+	+	27	+	ASSuT
K1	Chicken	2001	+	+	-	-	-	+	-	+	+	RDNC-b	+	-
K2	Chicken	2004	+	+	-	-	-	+	-	-	-	RDNC	+	-
K3	Chicken	2005	+	+	-	-	-	+	-	-	-	RDNC-c	+	-
K4	Chicken	2006	+	+	-	-	-	+	-	-	-	RDNC-c	+	-
K5	Chicken	2010	+	+	-	-	-	+	-	+	+	RDNC	+	ASuT
B1	Penguin	2009	+	+	+	+	+	+	-	+	+	RDNC	+	-
B2∼3	Crow	2000	+	+	+	+	+	+	-	+	+	RDNC-e	+	-
B4	Parrot	2005	+	+	+	+	+	+	-	+	+	RDNC-e	+	-
M1	Pork	2005	+	+	-	-	-	+	-	+	+	RDNC-a	+	-
M2	Pork	2007	+	+	-	-	-	+	-	+	+	RDNC-a	+	-
R1	River water	2007	+	+	+	+	+	+	-	+	+	26	+	-
R2	River water	2007	+	+	-	-	-	+	-	+	+	RDNC-a	+	ASu
R3	River water	2007	+	+	+	+	+	+	-	+	+	26	+	-

a m-PCR, multiplex PCR to identify *S*. Typhimurium [25]; IS*200*, PCR to identify *S*. Typhimurium [15]; up-*fljA*, boundary region of *fljA* and its upstream intergenic region; *fljA*–*fljB*, boundary region of *fljA* and *fljB*; *fljB*–*hin*, boundary region of *fljB* and *hin*; *hin*-down, boundary region of *hin* and its downstream intergenic region; +, positive; -,negative.

b +, presence; -, absence.

c RDNC, reacted but did not conform; RDNC-a–e, same letter indicates the same lysogenic patterns among RDNC isolates.

d LDC, lysine decarboxylase; +, positive; -, negative.

e A, ampicillin; S, streptomycin; Su, sulfamethizole; T, tetracycline; -, pansusceptible.

### Antimicrobial susceptibility testing

The Kirby–Bauer disc diffusion test was performed using Mueller–Hinton agar plates (Becton, Dickenson and Company) according to Clinical and Laboratory Standard Institute standards [23] using the following antimicrobials: ampicillin (10 µg), cefazolin (30 µg), kanamycin (30 µg), streptomycin (10 µg), tetracycline (30 µg), chloramphenicol (30 µg), fosfomycin (50 µg), colistin (10 µg), sulfamethizole (250 µg), and nalidixic acid (30 µg) (Becton, Dickinson and Company).

### Plasmid isolation

Plasmid DNA was isolated by the method described by Kado and Liu [24] followed by phenol–chloroform extraction. The Bac-Tracker Supercoiled DNA ladder (Epicentre Biotechnologies, Madison, WI, USA) and a 94-kb plasmid from *S*. Typhimurium LT2 were used as size markers.

### PCR and sequencing

All primers used in this study were purchased from Hokkaido System Science Co., Ltd. (Hokkaido, Japan) and are listed in Table S1. A single colony of each bacterial isolate was suspended in 50 µL of 25 mM NaOH and boiled for 5 min. After addition of 4 µL of 1 M Tris-HCl (pH 8.0), the suspension was centrifuged and the supernatant was used as a template DNA. Amplification was performed using an iCycler apparatus (Bio-Rad Laboratories, Hercules, CA, USA). Takara Ex Taq (Takara Bio Inc., Shiga, Japan) was used as DNA polymerase for each monoplex PCR. The *Salmonella* serovar Typhimurium Identification Kit (Takara Bio Inc.) was used to detect *S*. Typhimurium-related genes, including STM0292, STM2235, and STM4493, by multiplex PCR (m-PCR) as previously described [25]. Some of the PCR products were purified using the illustra ExoStar Kit for Enzymatic PCR and Sequencing Clean-up (GE Healthcare UK Ltd., Buckinghamshire, UK). Nucleotide sequences were determined on both strands using an Applied Biosystems 3130 xl genetic analyzer with the BigDye Terminator cycle sequencing kit (version 3.1; Applied Biosystems, Foster City, CA, USA). The sequences were assembled with Sequencher version 4 (Hitachi Solutions, Kanagawa, Japan) and the DNA alignments and deduced amino acid sequences were examined using the Basic Local Alignment Search Tool (http://blast.ncbi.nlm.nih.gov/Blast.cgi).

### Comparative genomic hybridization (CGH) analysis

Copy number analysis of the selected *S*. 4,[5],12:i:- isolates was performed using the whole genomic CGH array (Roche NimbleGen, Inc., Madison, WI, USA) at their facility in Iceland according to previously published methods with some modifications [26]. In brief, a tiling array was designed with a mean probe density of 1 probe/10 bp, 50–75-mer length using the *S*. Typhimurium strain LT2 sequences of the chromosome (AE006468) and pSLT plasmid (AE006471). Labeling was performed using the NimbleGen Dual Color Labeling Kit according to the manufacturer's protocols. In brief, each DNA sample (1 µg) was denatured at 98°C in the presence of one optical density of 5′-Cy3- or 5′-Cy5-labeled random nonamer. The denatured sample was chilled on ice and then incubated with 100 U of (exo-) Klenow fragment and dNTP mix for 2 h at 37°C. Reactions were terminated by addition of 0.5 M ethylenediaminetetraacetic acid (pH 8.0) and the end products were precipitated with isopropanol and resuspended in water. The Cy-labeled test and reference samples (Cy3 and Cy5, respectively) were combined (31 µg each) and dried down by vacuum centrifugation. Each sample was rehydrated in 5.6 µL of PCR grade water included in the NimbleGen Sample Tracking Control Kit and added to the hybridization buffers included in the NimbleGen Hybridization Buffer Kit, denatured at 95°C for 5 min, and then cooled to 42°C. Hybridizations were conducted for 40–72 h at 42°C using the NimbleGen Hybridization System. The arrays were washed using the NimbleGen Wash Buffer Kit and immediately dried down by centrifugation. Arrays were scanned at a resolution of 5 µm using the GenePix4000B scanner (Axon Instruments, Molecular Devices Corp., Sunnyvale, CA, USA). Data were extracted from scanned images using NimbleScan software (Roche NimbleGen, Inc.), which allows for automated grid alignment, extraction, and generation of data files.

### Gene replacement in *S*. Typhimurium

**Figure 1:**
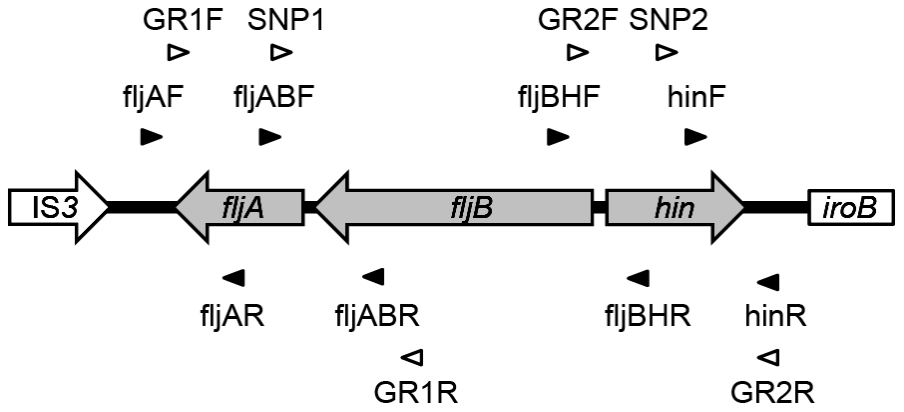
Schematic view of genetic organization of the chromosomal region related to flagellar phase variation of *S*. Typhimurium. Gray arrows indicate gene related to phase variation. Closed triangles indicate the primer locations for polymerase chain reaction mapping. Open triangles indicate the primer locations for mutant construction.

The primer pair GR1F and GR1R was used to amplify a region containing a point mutation in *fljA*, and the primer pair GR2F and GR2R was used to amplify a region containing a point mutation in *hin* from the *S*. 4,[5],12:i:- isolates by PCR (Fig. 1, Table S1). After digestion with the XbaI and HindIII restriction enzymes, the resulting fragment was cloned to the temperature-sensitive vector pTH18ks1 [27] and used as a vector for gene replacement. *S*. Typhimurium strains L-3900 and L-3287 were transformed with one of each vector by electroporation. The cells were spread on LB agar (Becton, Dickinson and Co.) supplemented with kanamycin and incubated at 28°C for 18 h. The colonies were then streaked on the same agar plates pre-warmed to 42°C and incubated at 42°C for 18 h. The single crossover strains were purified under the same conditions and then passaged at 28°C several times. The double crossover strains were screened by allele-specific PCR using the primer pair SNP1 and GR1R for detection of a point mutation in *fljA* and the primer pair SNP2 and GR2R for detection of a point mutation in *hin* (Fig. 1, Table S1). The introduced point mutation was verified by PCR and sequencing using appropriate primers. *S*. Typhimurium derivatives with the point mutation in *hin* were transformed with the vector for the *fljA* gene replacement. Double crossover strains were selected using the abovementioned procedure to obtain double mutants.

### Estimation of phase variation frequency

Phase variation frequency was estimated by the method described by Stocker [28] with minor modifications. In brief, the tested strains expressing the H-i antigen (phase 1) were serially passaged in LB broth media until the estimated number of generations reached 110. The culture was diluted to yield approximately 100 colonies on LB agar in 90-mm petri dishes and spread with a plastic spreader. The plates were incubated at 30°C for 18 h until the colonies grew to 1 mm in diameter. The plates were cooled in a refrigerator, and then 7 mL of semi-solid agar containing 0.35% Bacto Agar (Becton, Dickinson and Company) was pipetted on top. This semi-solid agar contained 0.7% (v/v) anti-H-i serum (Denka Seiken Co.). Once solidified, the plates were incubated at 37°C for 1–2 h. Colonies expressing the H-1,2 (phase 2) antigen were found to be surrounded by a wide zone of opacity with an indefinite edge, indicating that the organisms were swarming out into the semi-solid agar. However, colonies expressing the phase 1 antigen were surrounded by a narrow dense zone of opacity with a clear-cut edge. The frequencies of swarming colonies among ca. 7000 colonies were calculated, and then the frequency was divided by the number of generations to determine the frequency of phase variation per generation. Each experiment was performed thrice. Differences in the results were tested using the two-tailed unpaired Student's *t* test.

## Results and Discussion

### *S*. 4,[5],12:i:- is very likely a monophasic variant of *S*. Typhimurium

To prove the hypothesis that *S*. 4,[5],12:i:- is a monophasic variant of *S*. Typhimurium, several molecular characteristics of the *S*. 4,[5],12:i:- isolates were investigated. In the m-PCR to identify *S*. Typhimurium, three serovar-related genomic regions were successfully amplified from all the *S*. 4,[5],12:i:- isolates tested in this study (Table 1). No false positives were observed using 117 *Salmonella* serovars, with the exception of *S*. 4,[5],12:i:- [25], which strongly suggested that *S*. 4,[5],12:i:- originated from *S*. Typhimurium. The results of PCR analysis to detect the *fliA–fliB* intergenic region also support this statement. The location of IS*200* between the genes *fliA* and *fliB* can be used as a specific marker for *S*. Typhimurium [29]. The amplicon sizes from the *fliA–fliB* intergenic regions from *S*. Typhimurium and other serovars were expected to be 1000 and 250 bp, respectively [15]. A 1000-bp amplicon was successfully detected in all the *S*. 4,[5],12:i:- isolates. These data suggest that *S*. 4,[5],12:i:- is a monophasic variant of *S*. Typhimurium. In other words, the *S*. Typhimurium-specific m-PCR, and PCR might be useful to verify that the tested *S*. 4,[5],12:i:- isolate is a monophasic variant of *S*. Typhimurium.

As shown in Table 1, 39 (76.5%) of the 51 *S*. 4,[5],12:i:- isolates harbored a 94-kb plasmid with or without other plasmids. The *spvB* gene, which is a marker of the *S. enterica* virulence plasmid [30], was detected by PCR in all of the isolates with the 94-kb plasmid, thereby supporting the possibility that these isolates originated from *S*. Typhimurium. However, the lack of this plasmid does not contradict the possibility that the isolate is *S*. Typhimurium. The prevalence of the virulence plasmid in the *S*. Typhimurium isolates obtained from swine with systemic infections was 92%, whereas less than 20% in isolates from diarrhea samples and animals without any symptoms [31].

### Deletion is a major basis of the monophasic phenotype of the *S*. 4,[5],12:i:- isolates

Most *S. enterica* serovars possess two different flagellin proteins, including FliC (phase 1) and FljB (phase 2), which are encoded by the genes *fliC* and *fljB*, respectively. Flagellar phase variation is induced by inversion of the genetic region called the H segment, which contains the *hin* gene encoding for DNA invertase and the promoter for the *fljB* gene. The *fljB* constitutes an operon with the *fljA* gene, which encodes a negative regulator of *fliC* expression. FljA binds to the operator region of FliC mRNA and inhibits its translation, leading to the rapid degradation of FliC mRNA. When the H segment is in the “on” state, both *fljB* and *fljA* are transcribed, resulting in synthesis of phase 2 flagellin and inhibition of phase 1 flagellin. However, when the H segment is switched to the “off” state, neither *fljB* nor *fljA* are transcribed, resulting in the synthesis of phase 1 flagellin only (Fig. S1) [32], [33].

To determine whether the *S*. 4,[5],12:i:- isolates maintained the genetic structure of the *fljAB–hin* region, PCR mapping [7] to detect the boundary region of each gene was performed using the *S*. Typhimurium LT2 DNA sequence as a reference. The amplification targets were as follows; up-*fljA*, the boundary region of *fljA* and its upstream intergenic region; *fljA*–*fljB*, the boundary region of *fljA* and *fljB*; *fljB*–*hin*, the boundary region of *fljB* and *hin*; and *hin*-down, the boundary region of *hin* and its downstream intergenic region (Fig. 1). As shown in Table 1, three amplification patterns were observed: positive for only *hin*-down (31 isolates), all negative (12 isolates), and all positive (eight isolates). In eight of the positive isolates, the whole *fljAB*–*hin* structure was detectable with two common amino-acid substitutions: A46T in FljA and R140L in Hin. These amino-acid substitutions were not observed in six *S*. Typhimurium wild-type strains isolated in Japan including L-3900 and L-3287 (data not shown).

**Figure 2:**
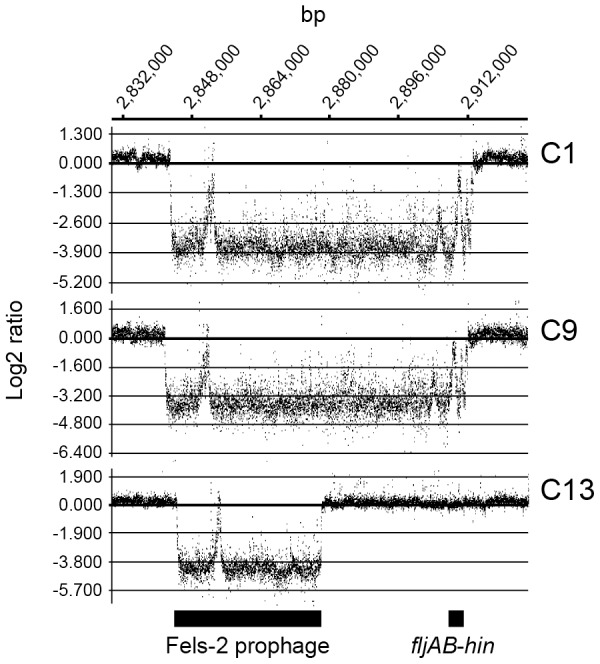
Partial quantitative data from the comparative genomic hybridization of the *S*. 4,[5],12:i:- isolates. The ruler indicates the nucleotide number of *S*. Typhimurium LT2 chromosome (AE006468). The vertical scale indicates the log2 ratio of the signal intensities. C1, C9, and C13 indicate the name of the isolates listed in Table 1. The underlying bold lines indicate the location of specific genetic structures in the chromosome.

To compare the whole genome sequences of the *S*. 4,[5],12:i:- isolates with that of *S*. Typhimurium strain LT2, one of the each representative isolate from the three amplification patterns determined by PCR mapping was analyzed by CGH. As shown in Table 2 and Fig. S2, whole sequences of prophages Fels-1 and Fels-2 were not detectable among the three isolates. In strains C1 and C9, an additional 36-kb sequence downstream of Fels-2 containing the *fljAB* operon was not detectable (Fig. 2). In addition, the whole sequence of the Gifsy-1 prophage was not detectable in strains C1 and C9, whereas part of the Gifsy-1 prophage sequence was not detected in strain C13 (Table 2 and Fig. S2). Among these, a broad scale deletion event stretching from the Fels-2 prophage to the *fljAB*–*hin* region was determined as the genetic basis of the monophasic phenotype of the *S*. 4,[5],12:i:- isolates C1 and C9.

**Table 2:** Chromosomal genes that lacks in the CGH tested isolates.

				Presence/Absence^a^		
NC_00319 tag number	Position (start-end)	Size (bp)	Description	C1	C9	C13
STM0276–STM0279	316895–319135	2241	putative cytoplasmic/periplasmic	+	+	-
			proteins			
STM0893–STM0929	962638–1005280	42643	Fels-1 prophage	-	-	-
STM1011–STM1019	1104868–1109917	5050	part of Gifsy-2 prophage	+	+	-
STM1555–STM1557	1632448–1635078	2631	putative Na^+^/H^+^ antiporter	+	+	-
			and others			
STM2585–STM2636	2730851–2776671	45821	Gifsy-1 prophage	-	-	-b
STM2694–STM2739	2844326–2877883	33558	Fels-2 prophage	-	-	-
STM2740–STM2771	2877884–2914231	36348	*fljAB* and upstream genes	-	-	+
STM2951	3094339–3096696	2358	*ygcF*	+	+	-
STM3113	3271613–3272493	881	*nupG*	-	-	+
STM3255–STM3260	3425101–3430141	5041	putative phosphotransferase system	-	-	+
			and others			

a Chromosomal genes with log2 ratios <−0.5 were identified as absent genes.

b Five genes (7782 bp) of the Gifsy-1 prophage were absent.

*S*. Typhimurium strain LT2 contains the DNA invertase gene *fin*, which contributes to the phase induction of H antigens other than *hin*. The *fin* gene is located in the Fels-2 prophage of *S*. Typhimurium LT2 [34]. All the *S*. 4,[5],12:i:- isolates were PCR-negative for *fin*, except for one isolate obtained from a swine, suggesting that the Fels-2 prophage was not distributed among most of the *S*. 4,[5],12:i:- isolates, as indicated by the CGH analysis of representative isolates.

In total, the presence/absence patterns of isolates C1 and C9 were identical, whereas strain C13 was different from other strains. Approximately 80% or more of the total length of the absence region corresponded to the Fels-1, Fels-2, Gifsy-1, and Gifsy-2 prophages (Table 2). Garaizar et al. [19] reported the deletion of most of the Fels-1 and Fels-2 sequences and a partial sequence of Gifsy-1 among the *S*. 4,[5],12:i:- isolates in Spain. Prophage sequences may be selectively neutral for this serovar.

### Point mutations reduce the phase variation frequency of *S*. Typhimurium

To manifest the effect of the amino acid substitutions, A46T in FljA and R140L in Hin observed in the eight *S*. 4,[5],12:i:- isolates by phase variation frequency analysis, these point mutations were independently or simultaneously introduced to *S*. Typhimurium strains L-3900 and L-3287. As shown in Table 3, phase variation was successfully observed in both parental strains and the frequency from phase 1 to phase 2 of L-3900 was 1.84×10^−4^, whereas that of L-3287 was less than that of the detection limit (10^−6^). The phase variation frequency of the L-3900 *fljA* mutant was significantly lower (*p* = 0.04) than that of the parental strain. An isolate expressing the phase 2 H antigen was successfully selected from this mutant, but not from any other mutants. These data suggest that the point mutations reduced the phase variation frequency and may be the genetic basis of the monophasic phenotype of all *fljAB*–*hin* detectable isolates.

**Table 3:** Expression of phase 2 antigen and phase variation frequency.

	Genotype^a^		Selectability	Phase variation
Parental strain	*fljA*	*hin*	of phase 2^b^	frequency^c^
L-3900	WT	WT	+	1.84×10^−4^
	A46T	WT	+	9.11×10^−5^ *
	WT	R140L	-	<10^−6^
	A46T	R140L	-	<10^−6^
L-3287	WT	WT	+	<10^−6^
	A46T	WT	-	ND
	WT	R140L	-	ND
	A46T	R140L	-	ND

a WT, wild type; A46T and R140L, amino acid substitutions.

b +, selectable; -, not detected.

c ND, not done;

^*^, significantly lower than parental strain (*p* = 0.04).

Hin invertase catalyzes DNA inversion of the H segment. This site-specific recombination event controls the alternate expression of two flagellin genes by reversing the orientation of the *fljB* promoter [32], [33]. The interaction between Hin invertase and the target DNA has been fully elucidated. The 52 carboxyl-terminal residues is the DNA-binding domain of the Hin invertase. Particularly, the sequence G^139^-R^140^-P^141^-R^142^ is essential to maintain DNA binding ability. The deletion of residues G^139^ and R^140^ abolished the sequence-specific binding to DNA [35]. The *fin* gene located in the Fels-2 prophage region encodes an invertase that can support inversion of the H segment without Hin invertase. As both the strains L-3900 and L-3287 were found to be negative for the *fin* gene by PCR, R140L in Hin may diminish the DNA-binding ability; thus, resulting in a reduction in the phase variation frequency. No information is available regarding the effect of the A46T substitution in FljA on the phase variation frequency to date.

### *S*. 4,5,12:i:- isolates in Japan consist of multiple distinct clones

As shown in Table 1, phage typing of the 51 *S*. 4,[5],12:i:- isolates examined using the *S*. Typhimurium typing phages identified four phage types: DT193 (eight isolates), DT26 (three isolates), DT27 (one isolate), and DT120 (one isolate). The remaining 34 isolates were RDNC (reacts with phages but does not confirm to a recognized pattern) and four isolates were UT (untypable). Among the 34 RDNC isolates, five lysogenic patterns were observed in more than one isolate, which were named RDNC-a–e. All eight isolates of lysine decarboxylase-negative *S*. 4,[5],12:i:- belonged to RDNC-a. In Germany and Switzerland, DT193 was the most prevalent definitive phage type among the *S*. 4,[5],12:i:- isolates [6], [17]. Most of the *S*. 4,[5],12:i:- isolates from Spain belonged to the definitive phage-type U302 [16]. The *S*. 4,[5],12:i:- isolates from Japan appeared to consist of multiple clones with greater variation than those from European countries.

Antimicrobial susceptibility testing using nine antimicrobials showed that 14 (27.5%) of the 51 *S*. 4,[5],12:i:- isolates were resistant to one or more antimicrobials among ampicillin, streptomycin, sulfamethizole, and tetracycline. The remaining 37 isolates were pan-susceptible (Table 1). According to the data published to date, most of the *S*. 4,[5],12:i:- isolates from Europe (i.e., Spain, Germany, and Switzerland) appeared to have a multidrug resistance phenotype, whereas most of the *S*. 4,[5],12:i:- isolates from North and South American countries (i.e., USA and Brazil) appeared to be pan-susceptible or resistant to only a few antimicrobials [6], [9], [16], [17]. In Japan, pan-susceptible isolates were dominant, although multidrug resistance isolates with resistance to up to four antimicrobials were detected. All of the six isolates obtained from swines exhibited resistance to multiple antimicrobials. This may reflect the extensive use of antibiotics as feed additives in the pig industry in Japan [36].

## Conclusions

The results of the molecular characterization of the 51 *S*. 4,[5],12:i:- isolates derived from various sources in Japan suggested that these isolates were very likely monophasic variants of *S*. Typhimurium. Deletion and point mutations were the bases of the monophasic phenotype of the *S*. 4,[5],12:i:- isolates. The results of phenotypic characterization suggested these isolates consisted of multiple distinct clones.

## Supporting Information

Figure S1 **A model for the molecular mechanism of phase variation in** ***Salmonella*** **cited from Yamamoto and Kutsukake [33]** **with slight modifications.** This system consists of two major parts: (i) the switching mechanisms of *fljB* promoter orientation by inversion of H segments and (ii) the FljA-mediated translational repression of *fliC* mRNA, leading to the rapid degradation of the mRNA. IR, inverted repeat; *fljBp*, *fljB* promoter; *fliCp*, *fliC* promoter; OP, operator region.
(TIF)

Figure S2 **Quantitative data from the comparative genomic hybridization of the** ***S*** **. 4,[5],12:i:- isolates.** The ruler indicates the nucleotide number of *S*. Typhimurium LT2 chromosome (AE006468). The vertical scale indicates the log2 ratio of the signal intensities. C1, C9, and C13 indicate the name of the isolates listed in Table 1. The underlying bold lines indicate the locations of prophages in the chromosome.
(TIF)

Table S1 **Primers used in this study.**
(DOCX)
