# Supplementary figures and images for: Characteristics of Salmonella enterica Serovar 4,[5],12:i:- as a Monophasic Variant of Serovar Typhimurium
Source: PLoS One. 2014 Aug 5;9(8):e104380. doi: 10.1371/journal.pone.0104380 (PMC4122451; doi:10.1371/journal.pone.0104380)

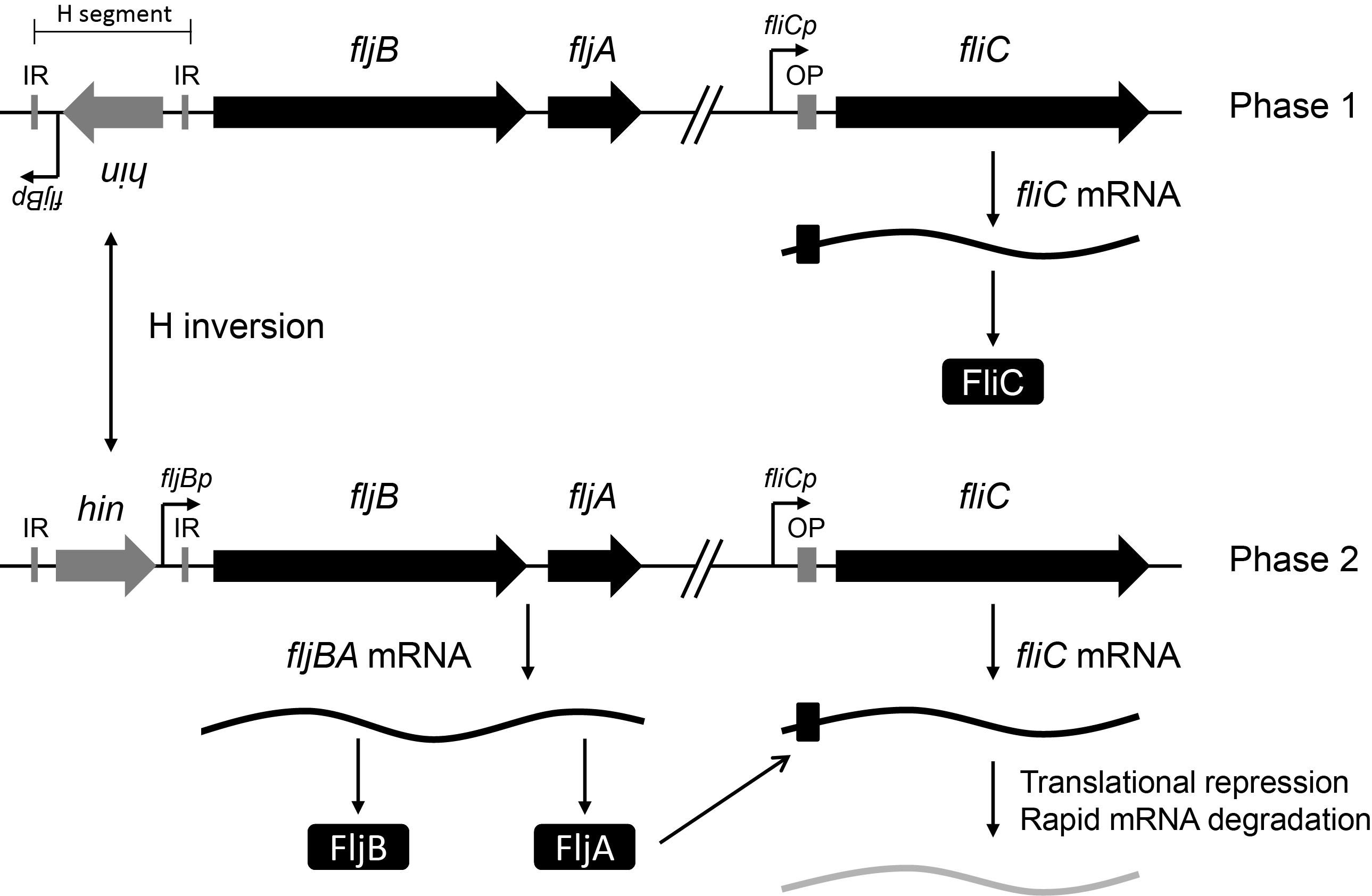

Supplement: Figure S1 — A model for the molecular mechanism of phase variation in Salmonella cited from Yamamoto and Kutsukake [33] with slight modifications. This system consists of two major parts: (i) the switching mechanisms of fljB promoter orientation by inversion of H segments and (ii) the FljA-mediated translational repression of fliC mRNA, leading to the rapid degradation of the mRNA. IR, inverted repeat; fljBp, fljB promoter; fliCp, fliC promoter; OP, operator region. (TIF) [file pone.0104380.s001.tif]

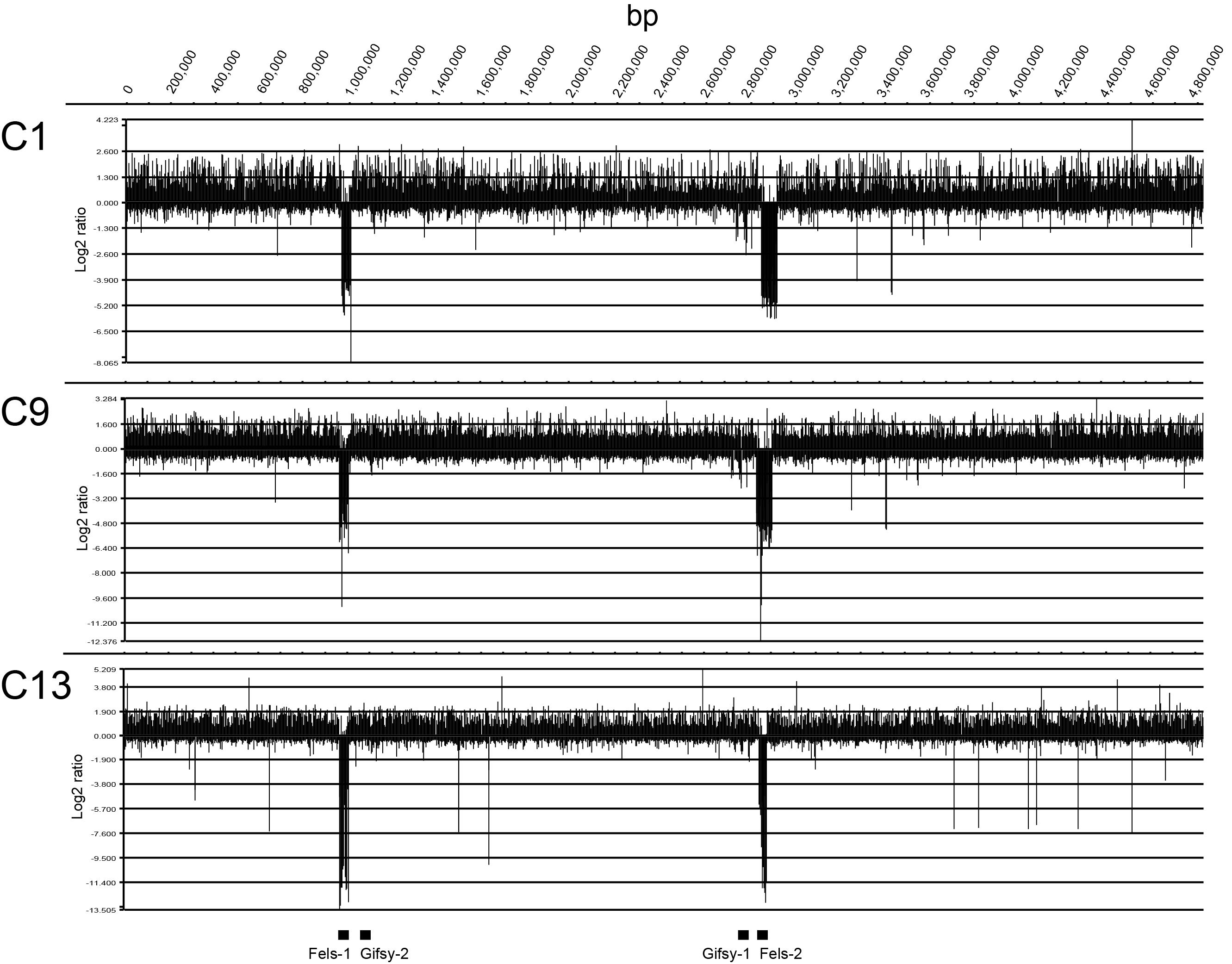

Supplement: Figure S2 — Quantitative data from the comparative genomic hybridization of the S . 4,[5],12:i:- isolates. The ruler indicates the nucleotide number of S. Typhimurium LT2 chromosome (AE006468). The vertical scale indicates the log2 ratio of the signal intensities. C1, C9, and C13 indicate the name of the isolates listed in Table 1. The underlying bold lines indicate the locations of prophages in the chromosome. (TIF) [file pone.0104380.s002.tif]
