# Supplementary material for: Characteristics of Salmonella enterica Serovar 4,[5],12:i:- as a Monophasic Variant of Serovar Typhimurium
Source: PLoS One. 2014 Aug 5;9(8):e104380. doi: 10.1371/journal.pone.0104380 (PMC4122451; doi:10.1371/journal.pone.0104380)
Supplement: Table S1 — Primers used in this study. (DOCX) [file pone.0104380.s003.docx]

Table S1. Primers used in this study

| Primer name | Sequence (5'→3')^a^ | Location | Reference |
| --- | --- | --- | --- |
| spvBF | AGCAGTTTTTATCGCCTGGA | *spvB* | This study |
| spvBR | GGTGGAACATCAGGACTTGG | *spvB* | This study |
| invAF | AAACCTAAAACCAGCAAAGG | STM2896 (*invA*) | 25 |
| invAR | TGTACCGTGGCATGTCTGAG | STM2897 (*invA*) | 25 |
| TSR1F | ATGCGGGTATGACAAACCCT | STM0292 | 25 |
| TSR1R | TTAGCCCCATTTGGACCTTT | STM0292 | 25 |
| TSR2F | CAGACCAGGTAAGTTTCTGG | STM2235 | 25 |
| TSR2R | CGCATATTTGGTGCAGAAAT | STM2235 | 25 |
| TSR3F | TTTACCTCAATGGCGGAACC | STM4493 | 25 |
| TSR3R | CCCAAAAGCTGGGTTAGCAA | STM4493 | 25 |
| FFLIB | CTGGCGACGATCTGTCGATG | *fliB* | 15 |
| RFLIA | GCGGTATACAGTGAATTCAC | *fliA* | 15 |
| finF | TTTGCTGTTTCGCCTAAACC | *fin* | This study |
| finR | CAACGCGACTGATTTACAGG | *fin* | This study |
| fljAF | TCCTGGACTGTTCCGGTATC | *IS3*-*fljA* intergenic region | 7 |
| fljAR | CGGCTGACGGATTTAATCTT | *fljA* | 7 |
| fljABF | CTTCGCAATGTAGCCTGTGA | *fljA* | 7 |
| fljABR | CTCTGATCTGGGTGCGGTA | *fljB* | 7 |
| fljBHF | GCAATGGAGATACCGTCGTT | *fljB* | 7 |
| fljBHR | TTAACGCCACCAGGTTTTTC | *hin* | 7 |
| hinF | GGCGATCAACAAACATGAAC | *hin* | 7 |
| hinR | ATGCCAGGCTTACCTGTGTC | *hin*-*iroB* intergenic region | 7 |
| GR1F | GCTCTAGACAAGAGTCTGGCACGATGAA (XbaI) | *IS3*-*fljA* intergenic region | This study |
| GR1R | CCCAAGCTTCTGACGGCACTACCAAAACA (HindIII) | *fljB* | This study |
| GR2F | GCTCTAGATCAGCAGCGACAGACTGTTA (XbaI) | *fljB* | This study |
| GR2R | CCCAAGCTTATGCCAGGCTTACCTGTGTC (HindIII) | *hin*-*iroB* intergenic region | This study |
| SNP1 | AGCCTGTGATTATCCGAGTA | *fljA* | This study |
| SNP2 | AAGGACGACTGGGAGGACTC | *hin* | This study |

^a^Restriction sites are underlined in the primer sequence and the names are identified in parentheses.
